# Effect of D-Limonene Nanoemulsion Edible Film on Banana (*Musa sapientum* Linn.) Post-Harvest Preservation

**DOI:** 10.3390/molecules27196157

**Published:** 2022-09-20

**Authors:** Chih-Yao Hou, Sulfath Hakkim Hazeena, Shu-Ling Hsieh, Bao-Hong Li, Min-Hung Chen, Ping-Yu Wang, Bao-Qing Zheng, Yu-Shen Liang

**Affiliations:** 1 Department of Seafood Science, National Kaohsiung University of Science and Technology, Kaohsiung 81157, Taiwan; 2 Department of Horticulture, National Chiayi University, Chiayi City 600355, Taiwan; 3 Yuan Marketing & Processing Division, Agriculture & Food Agency Council of Agriculture Executive, Nantou City 54044, Taiwan; 4 Department of Plant Industry, National Pingtung University of Science and Technology, Pingtung 912301, Taiwan

**Keywords:** D-limonene, nanoemulsion, fruit preservation, biocoating

## Abstract

D-limonene (4-isopropenyl-1-methylcyclohexene) is an important compound in several citrus essential oils (such as orange, lemon, tangerine, lime, and grapefruit). It has been used as a flavoring agent and as a food preservative agent, with generally recognized as safe (GRAS) status. D-limonene has been well-studied for its anti-inflammatory, antioxidant, anti-cancer, and antibacterial properties. The antibacterial activity of D-limonene against food-borne pathogens was investigated in this study by preparing a D-limonene nanoemulsion. The D-limonene solution and nanoemulsion have been prepared in six concentrations, 0.04%, 0.08%, 0.1%, 0.2%, 0.4%, and 0.8% (*v*/*v*), respectively, and the antibacterial activity was tested against four food-borne pathogens (*Staphylococcus aureus*, *Listeria monocytogenes*, *Salmonella enterica*, and *Escherichia coli*). The results showed that the D-limonene nanoemulsion had good nanoscale and overall particle size uniformity, and its particle size was about 3~5 nm. It has been found that the D-limonene solution and nanoemulsion have a minimal inhibitory concentration of 0.336 mg/mL, and that they could inhibit the growth of microorganisms efficiently. The data indicate that the D-limonene nanoemulsion has more antibacterial ability against microorganisms than the D-limonene essential oil. After bananas are treated with 1.0% and 1.5% D-limonene nanoemulsion coatings, the water loss of the bananas during storage and the percentage of weight loss are reduced, which can inhibit the activity of pectinase. The application of a biocoating provides a good degree of antibacterial activity and air and moisture barrier properties, which help with extending the shelf life of bananas.

## 1. Introduction

D-limonene (4-isopropenyl-1-methylcyclohexene) is an important constituent in several citrus essential oils (orange, lemon, tangerine, and grapefruit). It is considered generally recognized as safe (GRAS) by the United States Food and Drug Administration (FDA) and is widely used as a flavoring and food preservative agent [1]. It belongs to the family of terpenes and is readily volatile at room temperature. It is widely present in various citrus peels and essential oils, especially lemon oil, caramel oil, orange oil, bergamot oil, and dill oil. Limonene molecule contains a chiral center and can be seen in three different structural forms: -limonene, D-limonene, and a racemic form. D-limonene has a pleasant lemony taste, making it widely used as a flavoring agent and food additive in common foods such as fruit juice, candy, chewing gum, beverages, and ice cream. D-limonene is one of the most frequently used and cheapest fragrances in cosmetic formulations and can be found in many beauty products such as soaps, fragrances, shampoos, conditioners and body washes, cleaning products, and eco-friendly pesticides [2]. In addition, D-limonene is considered to be safe for food preservation [1] and can be used to extract natural green solvents [3]. After oral administration, D-limonene is rapidly absorbed, distributed, and metabolized by the gastrointestinal tract. D-limonene is considered safe, with low toxicity to humans, while not causing human carcinogenic or nephrotoxic risk [1].

Banana (family Musaceae and genus *Musa*), is rich in variety and has a wide range of uses, such as edible, medicinal, fiber, and ornamental purposes. Bananas, also known as plantains, are fragrant and nutritious fruits and can be harvested all year round. They are a rich source of vitamins and many biologically active compounds. They have a significant amount of dietary fiber and phenolic compounds and are one of the most commonly consumed fruits in the world [4]. The fruits can be classified according to the process of ripening, into climacteric fruits and non-climacteric fruits. During the ripening process of the climacteric fruits, the rate of respiration and the amount of ethylene produced will suddenly increase, forming a peak (i.e., catastrophic respiration), and then will fall back to the original value. Non-climacteric fruits have no such phenomenon, so their respiration rate is generally slower. The amount of ethylene produced is also very small, as it declines slowly as it matures. Ethylene can stimulate many biochemical reactions in climacteric fruits, make the fruit soft, produce an aroma, and decompose stored starch into sugar. The ripening process of climacteric fruit can continue after being picked, and ethylene can accelerate its ripening. The ripening of non-climacteric fruit will be interrupted after being harvested from the plant, and such fruit cannot continue to ripen. Ethylene does not promote ripening after harvesting in non-climacteric fruits, but it only accelerates its aging process, such as yellowing, odor, and reduced resistance to infection. Banana, as a climacteric fruit, has a relatively short shelf life after harvesting, due to its physiological characteristics [5].

In order to prolong the shelf life of fruits after harvest, various technologies are often used. Edible coatings, low temperature, low pressure, and controlled atmosphere storage have been applied to delay post-harvest ripening and the degradation of fruits and vegetables [6,7,8,9]. However, cold storage may cause cold and physiological damage to bananas [9] when stored in an environment below 13 °C. The peel will turn black, which will affect sales, and the cost of low pressure and controlled atmospheric storage is very expensive [8,10]. In recent years, edible coatings have been widely used in fruits and vegetables, such as cellulose, chitin, chitosan, etc. This is a highly economic and environmentally friendly food preservation technology. A semipermeable protective barrier that isolates water and vapor, thereby extending the shelf life of fruits and vegetables, has been used here.

D-limonene undergoes oxidative degradation under normal storage conditions, resulting in a loss of lemon flavor and the formation of off-flavors [11]. Its oxidation reaction leads to the formation of D-limonene hydroperoxide, which undergoes cleavage reactions to form alcohols, ketones, and epoxides [12]. In addition, its hydrophobicity is another disadvantage, due to the difficulty of achieving dispersion in water [13]. It requires the use of higher concentrations to achieve an equivalent antimicrobial efficacy in food systems. To alleviate the limitations of oxidation and hydrophobicity, many approaches have been explored to load D-limonene in different systems. Among them, nanoemulsions provide an efficient way to increase the stability of encapsulated bioactive compounds, thereby improving their antimicrobial activity in food matrices [14]. Nanoemulsions are emulsions with a very small droplet size of less than 100 nm [15,16]. The small particle size in nanoemulsions has two important characteristics. First, to enhance the stability of the physicochemical properties of the compounds therein; second, to improve the biological activity of lipophilic compounds by increasing the surface area per unit mass [17]. Nanoemulsions are increasingly being used to encapsulate, protect, and deliver lipophilic ingredients for food processing, especially of fruits, vegetables, etc. [18,19,20]. The same bioactive compounds encapsulated in nanoemulsions have a higher antibacterial activity than the conventional forms, due to their smaller droplet size. The resulting droplets may fuse with the bacterial cell wall, causing the pathogen’s lipid cell membrane to destabilize and cause bacterial death [21].

In this study, a D-limonene nanoemulsion was further made into an edible biocoating, which was applied to fruit preservation. A D-limonene nanoemulsion containing different concentrations was coated on bananas, and the biological properties during storage were measured. While analyzing pectin content and pectin lipase activity, the efficiency of antibacterial activity was detected. The ability of the nanoemulsion to act as a barrier to air and water vapor and to inhibit the growth of microorganisms and prevent the post-harvest ripening effect, thereby extending the shelf life of bananas, has been elucidated.

## 2. Results and Discussion

### 2.1. Microscopic Morphology of the D-Limonene Nanoemulsion

**Figure 1:**
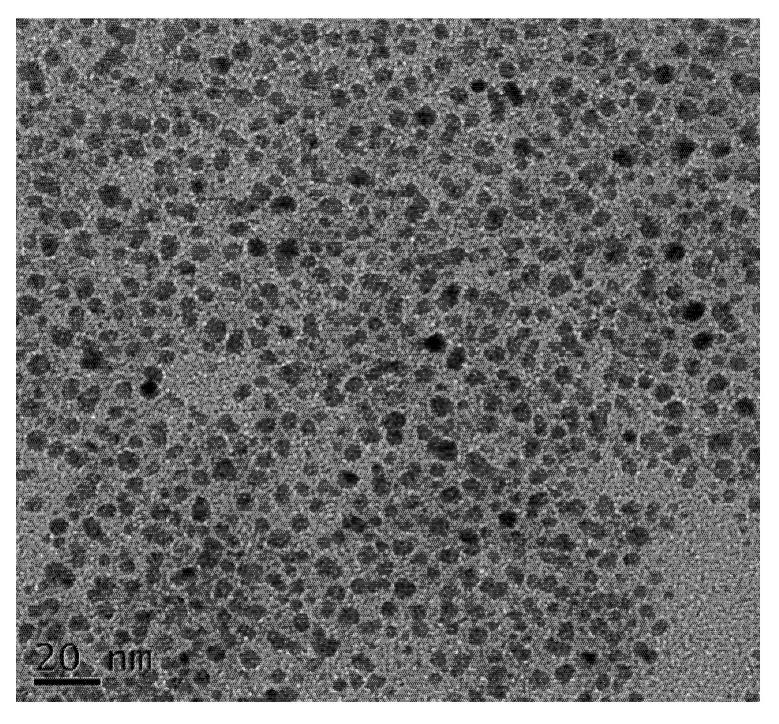
The size and shape of the D-limonene nanoemulsion droplets under transmission electron microscopy. Scale bar represents 20 nm.

After diluting the D-limonene nanoemulsion 20 times with deionized water, as commissioned by the Joint Center for High Valued instrument of National Sun Yat-sen University (Laboratory B2012, Second Experimental Building, College of Engineering) soft matter analysis was performed through transmission electron microscopy, to observe the particle size and shape of the nanoemulsion. As shown in Figure 1, the D-limonene nanoemulsion showed a uniform and spherical particle size of about 3~5 nm. The microscopic morphology indicates that the D-limonene nanoemulsion produced by the phase inversion method used in this experiment has good nanoscale and overall particle size uniformity, so it will be used in subsequent experiments.

### 2.2. Antibacterial Activity Results of the D-Limonene Nanoemulsion and D-Limonene Solution

#### 2.2.1. Antibacterial Activity of the D-Limonene Solution against Food-Borne Pathogens

Since D-limonene is difficult to dissolve in water, an appropriate surfactant should be used as the dilution solvent. Hence, 10% Tween 80 was used as the dilution solvent. D-limonene was prepared into six concentrations, 0.04%, 0.08%, 0.1%, 0.2%, 0.4%, and 0.8% (*v*/*v*), respectively, to simulate the antibacterial effects under different conditions. While checking the antibacterial activity, as shown in Table 1, the results show that the bacterial count of the *Staphylococcus aureus* control group reached 10.03 ± 0.01 log (CFU/mL). The inhibition situation of the 0.2% D-limonene group to *Staphylococcus aureus*, compared with the control group and sterile number growth, could achieve complete inhibition, indicating that D-limonene has a good bacteriostatic effect. The bacterial count in the *Listeria* control group was 9.73 ± 0.04 log (CFU/mL), and the 0.2% D-limonene group had an inhibitory effect on *Listeria* monocytogenes of up to 4.48 ± 0.06 log (CFU/mL). At about five logarithms, the bacterial count in the *Salmonella* control group was 10.14 ± 0.02 log (CFU/mL), and the 0.2% D-limonene group inhibited *Salmonella* by about two log values. *Escherichia coli* also had the same trend. *Escherichia coli* and *Salmonella* are both Gram-negative bacteria, while *Staphylococcus aureus* is Gram-positive. It can be seen from the table that the Gram-negative bacteria have a higher tolerance to 0.2% D-limonene, mainly because they have an extra layer of lipopolysaccharide (LPS) on the cell membrane compared to the Gram-positive bacteria. The LPS molecules in Gram-negative bacteria have unique permeability barrier properties, allowing the Gram-negative bacteria to exclude many toxic compounds, including drugs and antibiotics, and they can survive in harsh environments [22]. With the increase in D-limonene concentration, the group supplemented with 0.8% D-limonene achieved the complete inhibition of all four food-borne pathogens, which is consistent with the results presented by Wang et al. (2019).

#### 2.2.2. Effect of D-Limonene on the Antibacterial Activity of Food-Borne Pathogens after the Nanoemulsion Was Prepared

As shown in Table 2, the antibacterial activity of the D-limonene nanoemulsion was tested and compared with D-limonene solution. The limonene group had a bacterial count of 3.59 ± 0.12 log (CFU/mL) against *Staphylococcus aureus*, which was inhibited by about six log values compared to the control group. The *Listeria* control group had a bacterial count of 9.37 ± 0.10 log (CFU/mL), and the 0.1% D-limonene group had an inhibitory effect on the *Listeria* count of 2.39 ± 0.02 log (CFU/mL), an inhibition of about seven log values compared to the control group. The bacterial count of the *Salmonella* control group was 9.45 ± 0.05 log (CFU/mL), and the 0.1% D-limonene group had complete inhibition of *Salmonella* compared with the control group, so sterile growth was achieved. *Escherichia coli* also displayed the same trend. With an increasing concentration of D-limonene nanoemulsion, the 0.2% D-limonene nanoemulsion group achieved complete inhibition against four food-borne pathogens. Compared with the D-limonene solution, the nanoemulsion has better antibacterial activity, because the bioactive compound loaded in the nanoemulsion has a smaller droplet size than the conventional form. The resulting droplets may fuse with the bacterial cell wall, causing lipid cell membrane destabilization and cell damage [21].

#### 2.2.3. Minimal Inhibitory Concentration (MIC) and Minimal Bactericidal Concentration (MBC) of D-Limonene Solution and D-Limonene Nanoemulsion against Food-Borne Pathogens

The MIC (Minimal Inhibition Concentration) is the lowest concentration of a drug or agent that can inhibit the visible growth of bacteria. The MBC is the minimum concentration of an antimicrobial drug that is bactericidal. The two methods are arranged as shown in Table 3, and the MIC and MBC are expressed in mg/mL. 

Comparing the results of the two methods with each other, D-limonene was loaded into the nanoemulsion with the same concentration of D-limonene solution, and the results confirmed that the antibacterial activity could be improved by improving the permeability. Nanotechnology can be used to enhance the antibacterial activity of D-limonene [23,24]. The antibacterial mechanism of D-limonene is to irreversibly damage the cell membranes of microorganisms, resulting in the release of cellular components. Due to the increased surface area provided by the nanoemulsion and the high amount of contact, the ability of D-limonene to inhibit microorganisms will be improved in nanoemulsion form, compared to the solution [25].

### 2.3. Results of Quality Change of Bananas Treated with D-Limonene Nanoemulsion Coating

**Figure 2:**
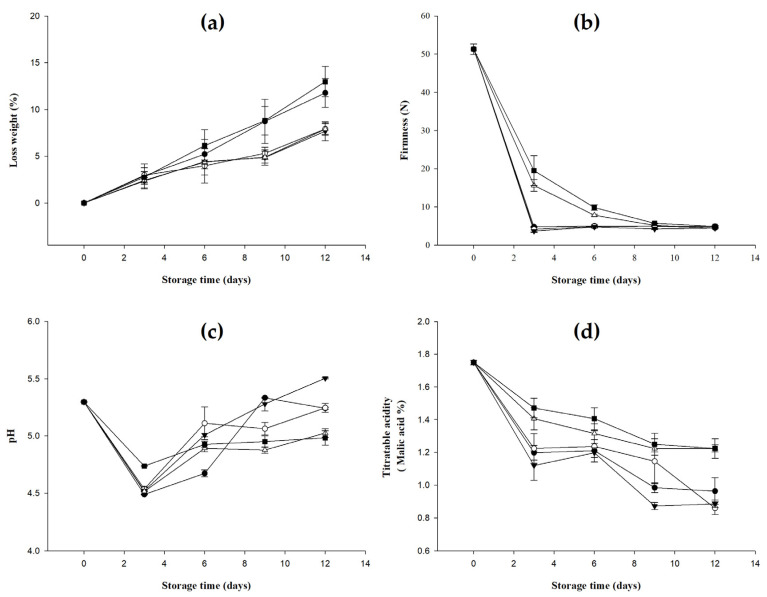
Effect on (**a**) weight loss, (**b**) firmness, (**c**) pH, and (**d**) titratable acidity (triplicate mean ± SD) of bananas coated with different concentrations of D-limonene nanoemulsion edible film and stored at 13 ± 1 °C from 0 to 12 days. (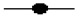) control, (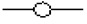) 0%, (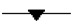) 0.5%, (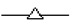) 1.0%, and (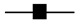) 1.5%.

Harvested fruits and vegetables easily lose moisture and weight, which reduces the quality and value of the fresh produce [26]. The bananas treated with a D-limonene nanoemulsion coating of different concentrations were stored for 0 to 12 days, and their weight loss was observed. As shown in Figure 2a, the percentage of the weight loss of bananas in each group showed an increasing trend during the storage period, and the increase in the control group and the 1.5% group was more obvious. Significantly different from the 0%, 0.5%, and 1.0% groups (*p* < 0.05) until the 12th day of the storage period, the weight loss of the two groups increased to 12.99 ± 1.60 and 11.77 ± 1.54%, respectively, which was about 4% higher than that of the other groups. The D-limonene nanoemulsion coating treatment could significantly reduce the loss of banana moisture and prolong its shelf life, but when the concentration of D-limonene nanoemulsion was too high (>1.5%), the epidermal structure of bananas was destroyed, and this accelerated the loss of moisture during storage.

Banana is a climacteric fruit and will continue to ripen during the storage period after harvesting. During the ripening period, it can be observed that the fruit firmness decreases. The decrease in firmness indicates the softening of the fruit, which is related to the dehydration and loss of the cell wall structure [27]. The results of firmness changes of bananas treated with D-limonene nanoemulsion coating are shown in Figure 2b for 0 to 12 days. The hardness was 51.30 ± 1.37 N on the 0th day, representing an immature banana. Its organization was firm and hard. In the control group and the 0% and 0.5% groups, the bananas ripened rapidly and the hardness dropped significantly to around 4 to 5 N on the third day of the storage period. Compared with the control group and the 0% and 0.5% groups, 1.0% of bananas in the 1.5% and 1.5% groups had a lower percentage reduction in firmness on the third and sixth days of storage, and there was no significant difference between the groups until the ninth day (*p* > 0.05). Bananas treated with an appropriate concentration of D-limonene nanoemulsion coating (1.0% and 1.5%) can better maintain hardness in the early stage of ripening, which means that the treatment can effectively slow down the metabolism and enzyme activity of bananas during the post-ripening period. In turn, the softening and degradation rate of the pulp tissue is slowed down.

During the ripening process of fruit, the contents of organic acids such as malic acid and tartaric acid will decrease, which will increase the pH value. The pH values of bananas treated with different concentrations of D-limonene nanoemulsion coating stored for 0 to 12 days are shown in Figure 2c. They began to show an upward trend after 12 days, and, on the 12th day, the control group, and the 0% and 0.5% groups had the highest increases, which were 5.24 ± 0.01, 5.24 ± 0.04, and 5.51 ± 0.01, respectively. An appropriate concentration of D-limonene nanoemulsion coating can inhibit the respiration and ripening of bananas during storage and reduce the change in pH value during the period.

With an increase in fruit maturity, the organic acid content in the fruit will decrease, and the sugar (soluble solids) content will increase [28]. Malic acid is the main organic acid in banana fruit [29]. The titratable acid changes of bananas treated with different concentrations of D-limonene nanoemulsion coating stored for 0 to 12 days are shown in Figure 2d. All the groups of bananas had significant titratable acid decreases (*p* < 0.05) from 1.75 ± 0.01% to 1.22 ± 0.06%, 1.22 ± 0.02%, 0.96 ± 0.08%, 0.89 ± 0.02%, and 0.86 ± 0.04%. This decrease was due to the organic acids (such as malic acid and lemon) in bananas, as they act as the substrates for the enzymatic reaction of fruit respiration [30]. The titratable acid decreased more slowly in the 1.0% and 1.5% groups during the storage period, indicating that the D-limonene nanoemulsion coating treatment can improve the shelf life and delay the aging of bananas.

### 2.4. Changes in Total Soluble Solids and Total Soluble Sugars of Bananas Treated with D-Limonene Nanoemulsion Coating

**Figure 3:**
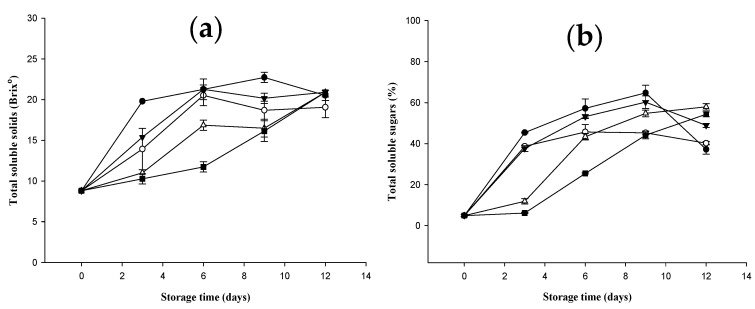
Effect on (**a**) total soluble solids and (**b**) total soluble sugars (triplicate mean ± SD) of banana coated with different concentrations of D-limonene nanoemulsion edible film, and stored at 13 ± 1 °C from 0 to 12 days. (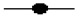) control, (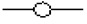) 0%, (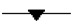) 0.5%, (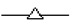) 1.0%, and (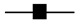) 1.5%.

The total soluble solids (TSS) of bananas treated with different concentrations of D-limonene nanoemulsion coating, stored for 0 to 12 days, are shown in Figure 3a. Within the first 6 days of the storage period, the TSS (Brix) content in the banana fruit showed an increasing trend. The control group and the 0% and 0.5% groups had TSS contents of 21.27 ± 1.27, 20.53 ± 1.27, and 21.27 ± 1.27 on the sixth day of storage, respectively. The higher increase in TSS content may be related to the degradation of starch and the dehydration of the pulp during storage, while the increase in the 1.0% and 1.5% groups was slower, reaching 16.86 ± 0.64 and 11.73 ± 0.64, respectively. The slower increase in TSS in the nanoemulsion-coated fruit may be due to the inhibition of gas exchange, which leads to the slowing down of fruit metabolism [31]. Similar trends were found in previous studies using biopolymer coatings on fruits [26,32], but the control and the 0% and 0.5% groups showed increased TSS after day 9 of the storage period. The content showed a trend of slowing down, probably due to the over-ripening of bananas. Increased glycolysis resulted in a decrease in TSS content, while the 1.0% and 1.5% groups continued to increase, indicating that the fruit was still in the ripening stage.

The total soluble sugars of bananas coated with D-limonene nanoemulsion at different concentrations was stored for 0 to 12 days, and the change results of the total soluble sugars are shown in Figure 3b. Total soluble sugars are a good indicator of fruit ripening, because stored starch will be hydrolyzed into total soluble sugars during the ripening process [33]. On the ninth day of the storage period, the total soluble sugars in all groups (%) increased, which was most obvious in the control and the 0% and 0.5% groups. On the third day of the storage period, the total soluble sugars increased from the initial 4.93 ± 0.54% to 45.44 ± 0.24%, 38.73 ± 0.67%, and 37.63 ± 1.42%, while the 1.0% and 1.5% groups slowly increased to 11.89 ± 1.25% and 6.15 ± 0.27%, respectively. The reason for this may be that the appropriate concentration of D-limonene nanoemulsion coating can block the contact between banana and air, slowing down the respiration rate of bananas after ripening and thereby reducing the hydrolysis of starch. After the ninth day of the storage period, the total soluble sugar content of the control group and the 0% and 0.5% groups showed a trend of slowing down and decreasing, which may be due to the glycolysis of bananas from excessive ripening. The 1.0% and 1.5% groups continued to increase, rising to 57.99 ± 1.59% and 54.34 ± 1.21% on the 12th day of storage, respectively, and the trend was consistent with the change in total soluble solids.

### 2.5. Results of Microbial Changes in Bananas Treated with D-Limonene Nanoemulsion Coating 

**Figure 4:**
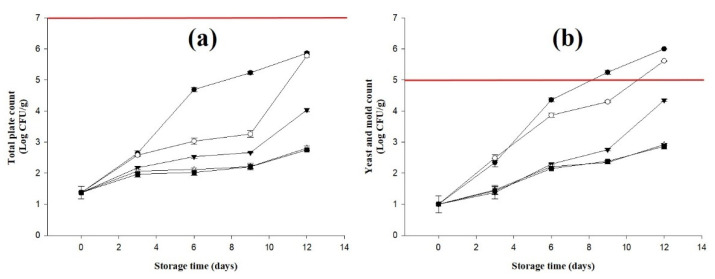
Effect on (**a**) total plate count and (**b**) total yeast and mold count (triplicate mean ± SD) of bananas coated with different concentrations of D-limonene nanoemulsion edible film and stored at 13 ± 1 °C from 0 to 12 days. (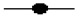) control, (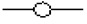) 0%, (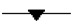) 0.5%, (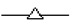) 1.0%, and (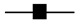) 1.5%. Red line indicates the critical limit for human consumption.

Bananas contain high amounts of water, starch, and other vitamins and are an excellent source of nutrients for microbial growth indicator [30,34]. The results of the total microbial count of bananas treated with D-limonene nanoemulsion coating after storage for 0 to 12 days, including total plate count and total yeast and mold count, are shown in Figure 4.

The number of bacteria in each group increased with time, and the control group and the 0% group had the most obvious increase, from the initial 1.38 ± 0.21 log (CFU/g) up to 5.86 ± 0.03 log (CFU/g) and 5.78 ± 0.04 log (CFU/g), respectively, on day 12 of the storage period. The 0.5% group also rose to 4.04 ± 0.06 log (CFU/g), while the 1.0% and 1.5% groups slowly increased to 2.81 ± 0.07 log (CFU/g) and 2.75 ± 0.03 log (CFU/g), respectively, which was suppressed more strongly by three log values compared to the control group and the 0% group. This result indicates that the D-limonene nanoemulsion coating can inhibit the growth of microorganisms in bananas and that it has very high antibacterial activity at concentrations of 1.0% and 1.5%. During the storage period, it has a high inhibitory effect on the growth of microorganisms, but it will cause dark brown lines on the surface of bananas and affect their appearance.

The trend of total yeast and mold counts during the storage period was consistent with the results of total bacterial counts. The bacterial counts of the control group and the 0% group increased to 6.00 ± 0.02 log (CFU/g) and 5.78 ± 5.78 ± 1.5% on the 12th day of the storage period, respectively, and 0.04 log (CFU/g), which exceeded the critical limit of total yeast and mold count in fruits and vegetables during storage. The critical limits of total bacterial count and total yeast and mold count are 10^8^ CFU/g and 10^5^ CFU/g, respectively [35]. The 1.0% and 1.5% groups were both lower than 3 log (CFU/g), indicating that the D-limonene nanoemulsion coating had good antibacterial and antifungal effects. Using the appropriate concentration of D-limonene nanoemulsion coating, bananas can maintain their quality during storage, inhibit the growth of microorganisms, and prolong shelf life.

### 2.6. Changes in Pectinase Activity and the Total Pectin Content of Bananas Treated with D-Limonene Nanoemulsion Coating

**Figure 5:**
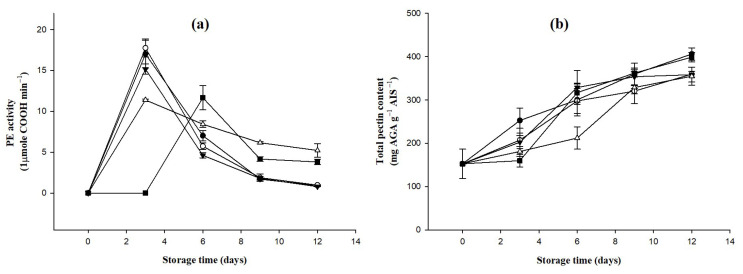
Effect on (**a**) PE activity and (**b**) total pectin content (triplicate mean ± SD) of bananas coated with different concentrations of D-limonene nanoemulsion edible film and stored at 13 ± 1 °C from 0 to 12 days. (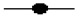) control, (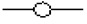) 0%, (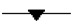) 0.5%, (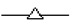) 1.0%, and (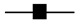) 1.5%.

During different growth periods of fruits and vegetables, the activity of pectinase will vary. For example, after the banana is harvested, the activity will increase when the peel is green to yellow, but if it is overripe and reaches the aging stage, the activity will decrease [36]. Figure 5a shows the results of changes in the pectinase activities of bananas treated with D-limonene nanoemulsion coating at different concentrations from 0 to 12 days. In the 0% control group and the 0.5% and 1.5% groups, the pectinase activity of bananas increased with time within 3 days of storage and then showed a downward trend. Units rose to 17.02 ± 1.70, 17.76 ± 1.11, and 15.17 ± 0.64, respectively; the 1.0% group increased at a lower rate of 11.40 ± 0.13 units and then the rate of decrease was slower, indicating that it could inhibit the production of pectinase. In the 1.5% group, the activity of pectinase was not observed in the first 3 days of the storage period, and the activity only increased to 11.67 ± 1.49 units on the sixth day and then began to decrease. This means that bananas treated with an appropriate concentration (1.0%) of D-limonene nanoemulsion coating can delay their ripening effect after harvesting and have an inhibitory effect on pectinase, compared to those treated with a higher concentration (1.5%) of D-limonene. In bananas treated with limonene nanoemulsion coating, the production of pectinase was delayed for 3 days, and it did not adversely affect the tendency of pectinase activity during the post-ripening process of the bananas.

The softening of the pulp during fruit ripening is associated with changes in cell wall structure [37]; these changes include an increased solubility of cell wall pectins, involving the actions of cell wall hydrolases, polygalacturonase, pectin esterase, β-galactosidase, and cellulases [38]. Under the influence of pectinase, the contents and properties of pectin will change with the after-ripening of bananas, in which protopectin will be transformed into pectin and, finally, into pectic acid [39]. The total pectin contents of bananas treated with different concentrations of D-limonene nanoemulsion coating and stored for 0 to 12 days are shown in Figure 5b. The total pectin content in each group of bananas increased with time. The increase was most obvious in the control group and in the 0% and 0.5% groups on the third day of the storage period, from 152.62 ± 33.76 mg/g to 252.62 ± 28.87, 207.38 ± 27.04, and 202.62 ± 14.87 mg/g, respectively. For the 1.5% group on the third day, although the total pectin content was only 159.76 ± 14.87 mg/g, it increased sharply on day 6 to 316.90 ± 21.82 mg/g, which was not significantly different from the control group and the 0% and 0.5% groups (*p* > 0.05), while the 1.0% group slowly increased to 212.14 ± 25.75 mg/g until the storage period. It only increased to 328 ± 36.65 mg/g on the ninth day, and this result showed a positive correlation with the change in pectinase activity, indicating that the application of D-limonene nanoemulsion coating with an appropriate concentration on bananas can delay the growth of the banana post-ripening process.

### 2.7. Changes in the Color Characteristics of Bananas Treated with D-Limonene Nanoemulsion Coating

Color is an important quality attribute of fresh fruits and vegetables [40], and it plays a key role in consumer preference and the acceptability of food. By measuring L* (brightness), a* (red-green), and b* (yellow-blue) values, the color properties of bananas during storage have observed. The changes in the color characteristics of bananas treated with D-limonene nanoemulsion coating at different concentrations stored for 0 to 12 days have been noted, and the results are shown in Table 4. For the L* value, the difference between the control group and the 0%, 0.5%, and 1.0% groups for all bananas increased with time, probably due to the yellowing of bananas during ripening, which increased the brightness. With the increase in storage time, each group showed a downward trend, because the surfaces of the bananas began to brown after over-ripening. The appearance of black spots occurred, among which the bananas in the 1.0% group retained the highest value (54.32 ± 0.78) on the 12th day of storage, while the bananas in the 1.5% group showed a sharp decrease in L* value throughout the storage period. The trend decreased from an initial 58.25 ± 0.52 to 42.80 ± 0.28 on the 12th day of storage, because the high concentration of D-limonene nanoemulsion coating will destroy the bananas’ surface, produce a dark texture, and continue to affect the bananas’ surface color. For the a* value, the values of the coated bananas had obvious changes, and the bananas in the 1.5% group had the lowest value during the storage period, from an initial 5.30 ± 0.00 to the 12th day of the storage period. The day time decreased to −3.38 ± 1.23, indicating that the bananas’ surface still retained an unripe green color during the storage period. In the part of the b* value, the control group and the 0%, 0.5%, and 1.0% groups increased roughly with time, which was due to the change of the color of the bananas from green to yellow during the ripening process. With an increase in storage time, all groups showed a downward trend, and the bananas in the 1.5% group had the most obvious decline, from 45.74 ± 1.35 at the beginning to 26.18 ± 4.55 on the 12th day of storage. The concentration of the D-limonene nanoemulsion coating and the resulting dark texture will affect the interpretation of surface color.

### 2.8. Changes in the Appearance of Bananas Treated with D-Limonene Nanoemulsion Coating 

During the post-ripening period of bananas, the fruit will turn from green to yellow. In the yellow stage, the fruit will continue to ripen, and polyphenol oxidase accumulates. The presence of PPO further catalyzes the hydrolysis of phenolic compounds into O-quinone, which is then oxidized and polymerized into dark brown melanin. Shellac and gelatin composite films were used to develop edible surface coatings for the shelf-life extension of banana fruits. The physicochemical properties and film coating efficiencies of composite films with different concentrations of gelatin were also evaluated for viscosity, surface tension, pH, work of adhesion, and spreading coefficient. The results indicated that an increase in gelatin content had an effect on the changes in the work of the adhesion and spreading coefficient, and, hence, the film coating efficiency over a banana skin significantly changed. Among all the concentrations of gelatin, 40% gelatin exhibited the appropriate concentration for application as an edible film coating. Therefore, the composite film based on 60% shellac and 40% gelatin, with and without PEG 400 at 5%, as the edible coating solution for extending the shelf life of bananas, was further investigated. The coating method of the composite film over bananas was prepared using a dipping technique. The temperature of all of the coated samples was kept stable at 25 °C for 30 days. Changes in the physicochemical and microbiological properties, such as color, titratable acidity, total soluble sugar, weight loss, firmness, and total yeast and mold counts were also investigated. Composite films, with and without PEG 400, can be applied as an edible coating for post-harvest fruits, and they have beneficial effects on retarding the ripening process. They act as an effective physical barrier around the fruit, resulting in slow decreases in weight loss, softening, the amount of acid and sugar, and maintaining the quality of post-harvest fruits for more than 30 days, compared with uncoated fruits. Therefore, a composite film based on shellac and gelatin could be utilized, adding to the value of fruits and vegetables by prolonging their post-harvest shelf life. The changes in the appearance of bananas treated with different concentrations of D-limonene nanoemulsion coating for 0 to 12 days are shown in Table 5. In the control group, it can be seen that the bananas began to change to yellow significantly from the third to the sixth day during the storage period. Later they began to produce black physiological spots, which can be clearly observed on the ninth day, with the highest proportion on the 12th day, almost covering the entire banana. The bananas in the 0.5% group also showed the same trend. This amount was slightly lower than that of the control group. After the bananas in the 1.5% group were treated with D-limonene nanoemulsion coating, they developed dark textures on the third day of storage, and their color became more obvious with time. The phenomenon of banana shrinkage was observed on the sixth day of the storage period. The bananas of the 0% and 1.0% groups were less ripe than the bananas of the other groups and still retained their unripe green parts. During the entire storage period, the bananas’ surface did not produce browning and black physiological spots, which means that this can delay the ripening effect of the bananas, which is beneficial to the quality of bananas after harvesting and prolonging their shelf life.

### 2.9. Microscopic Morphologies of D-Limonene Nanoemulsion Coatings on Banana Surfaces with Different Concentrations

**Figure 6:**
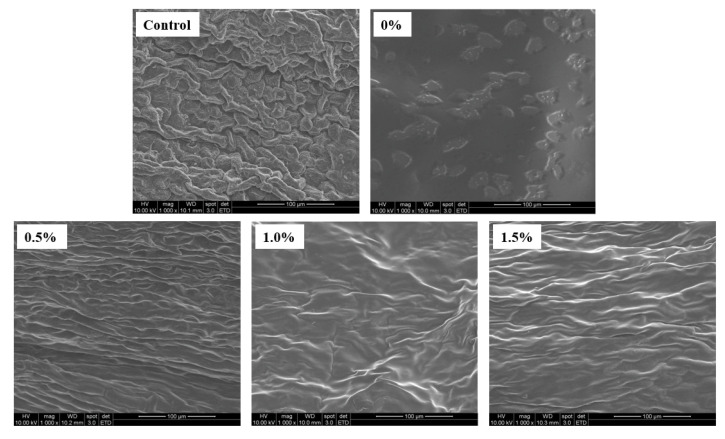
Surface morphological properties of bananas coated with different concentrations of D-limonene nanoemulsion edible film. Scale bar represents 100 μm.

For the D-limonene nanoemulsions with different concentrations on bananas, the Guiyi Center of National Sun Yat-sen University (Laboratory A3019, Huan Gong Building and College of Engineering) was entrusted to conduct environmental scanning electron microscope photography to observe the morphology of the bananas’ surface, as shown in Figure 6. The surface of the untreated banana (control group) presented an irregular concavo-convex shape, and the coating of the 0.5%, 1.0%, and 1.5% groups completely covered the surface of the banana without the phenomenon of gaps, forming a good barrier. The protective layer between the bananas’ surface and the outside environment in the 0% group completely covered the bananas’ surface. However, in terms of morphology, due to the high thickness of the coating itself, only a slight unevenness on the bananas’ surface could be observed. From an analysis of the experimental results, a coating could be observed that completely covered the bananas’ surface. Although the coating thickness of the 0% group was higher, it had no effective effect on inhibiting the ripening of the banana. It can be concluded that D-limonene nanoemulsion was a key ingredient in inhibiting the post-ripening effect of bananas.

## 3. Materials and Methods

### 3.1. Materials

D-limonene (97%) were purchased from Alfa Aesar (Tewksbury, MA USA). Plate count agar and Tryptone soy broth were purchased from Oxoid (Basingstoke, Hampshire, United Kingdom). Ethanol (95%) was purchased from Echo Chemical Co. (Miaoli County, Taiwan). Acetone were purchased from Seedchem (Box Hill, VIC, Australia). Sulfuric acid was purchased from Aencore Chemical Co. (Box Hill, VIC, Australia). Sodium chloride, disodium hydrogen phosphate, sodium hydroxide, and sodium tetraborate were purchased from Showa Chemical Industry Co. (Minato-ku, Tokyo, Japan). Potassium dihydrogen phosphate were purchased from Katayama Chemical Industries Co. (Hyogo, Japan). Propylene glycol were purchased from JT Baker (Phillipsburg, NJ, USA). Tween 80, glucose, phenol, and pectin (from citrus peel) were purchased from Sigma Aldrich (Quentin Fallavier, France). The 3-Phenylphenol and sodium alginate were purchased from Acros organics (Trenton, NJ, USA). Potassium hydrogen phthalate was purchased from Fullin Nihon Shiyaku (Taoyuan, Taiwan). Dichloran rose Bengal chloramphenicol agar was purchased from Becton, Dickinson and Co. (Washington, DC, USA).

### 3.2. Methods

#### 3.2.1. Preparation of D-Limonene Nanoemulsion Coating

D-limonene nanoemulsion coating was prepared with some modifications by Yanan Li et al. [41]. Deionized water and propylene glycol were mixed uniformly to prepare an aqueous phase with a volume ratio of 2:1. The oil phase was composed of D-limonene and 6% Tween 80 at mixed at different concentrations. The aqueous phase and the oil phase were mixed by slowly adding the water phase to the oil phase at a stirring rate of 800 rpm and keeping the addition rate constant, at 1.0 mL/min. After the addition of the aqueous phase, the stirring continued for 6 h to form an oil in water nanoemulsion. A 1% sodium alginate solution was added to the emulsion and stirred evenly, and the result was used as a D-limonene nanoemulsion coating for further experiments.

#### 3.2.2. Transmission Electron Microscopic (TEM) Analysis of D-Limonene Nanoemulsion

A volume of 10 μL of D-limonene nanoemulsion diluted 20 times was used to cover the carbon-plated copper mesh for TEM. The test piece was dried via vacuum drying and a soft matter analysis transmission electron microscope (JEOL, JEM-2100) was used to observe the microscopic morphology and size of the D-limonene nanoemulsion. From the scale bar provided in the microscopic images, the particle size was calculated.

#### 3.2.3. Antibacterial Activity of D-Limonene Nanoemulsion and D-Limonene Solution

##### Strain Activation

In this study, four food-borne pathogens (*Staphylococcus aureus* ATCC 25923, *Listeria monocytogenes* ATCC 19111, *Salmonella enterica* ATCC 13076, and *Escherichia coli* ATCC 23815) were cultured. The standard strains were taken out from −80 °C (contains the bead strain preservation tube), inoculated into an appropriate amount of TSB culture solution, and cultured with shaking at 37 °C for 16 h. After activation, the bacteria were streaked onto the TSA medium for a follow-up experiment.

##### Antibacterial Test (Broth Dilution Method)

D-limonene nanoemulsion with different concentrations was prepared by mixing 10% Tween 80 and D-limonene solution. The concentrations were 0.8%, 0.4%, 0.2%, 0.1%, 0.08%, and 0.04% (*v*/*v*) as well as a negative control (without D-limonene). The cultured bacterial solution was diluted to 10^6^ CFU/mL via serial dilution, and 50 μL of the diluted bacterial solution was mixed with 1000 μL of both D-limonene solution and D-limonene nanoemulsion of different concentrations in a 5 mL sterile centrifuge tube. The mixture was poured into 9 mL of TSA culture solution sterilized previously and incubated at 37 °C with shaking for 16 h. The grown bacterial suspension was diluted 10^−1^~10^−9^ times using the serial dilution method. Later, 1 mL of each diluent was mixed with the PCA medium via a mixing method and kept inverted for 24 h at 37 °C to calculate the number of colonies.

#### 3.2.4. D-Limonene Nanoemulsion Coating Used for Banana Fruit Preservation

Bananas were purchased from Qishan District, Kaohsiung City. Fresh bananas with uniform color, shape, and maturity were selected. Their surfaces were cleaned and coated with different concentrations of D-limonene nanoemulsion coating (0%, 0.5%, 1%, 1.5%, and control) and air-dried. The process of coating treatment involved preparing a quantitative coating solution in advance and using a brush to evenly coat the fruit. During the coating process, the coating solution dripped from the fruit was recovered in the beaker. The volume coated on the banana fruit was the volume of the original quantification minus the remaining volume and the recovered volume. At the same time, the volume of coating solution per square centimeter of the fruit surface can be calculated. In the study, the average volume of the coating solution on banana peel was about 4.6~6.2 μL/cm^2^. After coating treatment, the fruit was stored at 13 ± 1 °C for a 12-day storage test and analysis, performed at 0, 3, 6, 9, and 12 days.

#### 3.2.5. Physical and Chemical Analysis

##### Weight Loss

A digital balance (AP 224X, Shimadzu, Japan) was used to measure the weight of the sample on the days of analysis, and the weight loss percentage was calculated using the following formula.

Weight loss Δm (%) = (m_i_ − m_f_)/m_i_ × 100 (1)

where m_i_ = the initial mass of the sample, and m_f_ = the mass of the sample in days of analysis.

##### Firmness

Using a physical property analyzer (CR-500DX, Tokyo, Japan), the firmness of the banana sample was analyzed. A round probe with a diameter of 10 mm was used to test the hardness of the banana. The firmness analysis condition was 100 mm/min, and the penetration depth was 5 mm.

##### pH 

In total, 20 g of banana sample and 80 mL of deionized water were added and homogenized. Using a pH meter (SP-2500, SUNTEX, Taipei, Taiwan), the pH of the sample was checked.

##### Total Soluble Solids (TSS)

Banana pulp (20 g) and 80 mL of deionized water were mixed and homogenized with a homogenizer (Simple blend10, Oster, TX, USA), and the mixture was filtered with filter paper (Advantec paper No. 1). The TSS in the filtrate was tested using a handheld refractometer (Master-BX/S28 M, ATAGO, Tokyo, Japan).

##### Titratable Acidity (TA)

The filtrate was titrated for the determination of total soluble solids with 0.1 N NaOH to pH 8.3, and the titratable acidity was calculated using the following formula. The result is expressed as the percentage of malic acid equivalent, which is the main acid present in ripe bananas.

Titratable acidity (%) = V × 0.1 × F × 0.0067/W × 100(2)

where V = NaOH titration (mL), F = 0.1 N NaOH titer, and W = banana sample weight (g).

##### Total Soluble Sugars

The total soluble sugars were analyzed by adding 0.2 g of sample to 10 mL of 80% ethanol and incubating it in a water bath at 80 °C for 30 min. The supernatant was diluted, 400 μL was added to 200 μL of 5% phenol, and 1000 μL concentrated sulfuric acid was added for color development. After cooling, the absorbance was measured at a wavelength of 480 nm, and a glucose solution (0~100 μg/mL) was used as the standard solution for calculating the total soluble sugar content. 

#### 3.2.6. Microbiological Analysis

A total of 20 g of each sample was homogenized in 180 mL of sterile phosphate buffer saline (PBS) solution; serial dilution was performed, and 1 mL of each was poured into Petri plates. Plate count agar and Dichloran rose Bengal chloramphenicol agar were poured later for total plate and yeast and mold count, respectively. The entire experiment was performed under sterilized conditions. Total plate count plates were incubated in a BOD maintained at 37 °C for 48 h, while the total yeast and mold count plates were incubated at 25 °C for 72 h.

#### 3.2.7. Pectin Analysis

##### Total Pectin Content Determination

Total pectin content was determined according to Hou et al. (2008) [42]. Precooled H_2_SO_4_ (2 mL) and distilled water (15 mL) were slowly added to 5 mg alcohol insoluble solid (AIS) of banana. The mixture was filtered with filter paper to obtain a pectin solution after magnetic stirring for 1 h in an ice bath. An adequate volume (0.5 mL) of the pectin solution was mixed with 3 mL of 12.5 mM sodium tetraborate solution (in sulfuric acid) in an ice bath and then heated in a boiling water bath for 5 min. After cooling, the reaction mixture was mixed well with 0.05 mL of 0.15% m-hydroxydiphenyl solution in 0.5% NaOH, and then rested for 5 min. The absorbance at 520 nm was recorded. D-galacturonic acid was used to construct the standard curve for the calculation of pectin content in the sample.

##### Pectin Esterase (PE) Activity Determination

After peeling the banana, its pulp was taken, and four lots of a 2% NaCl solution (*w*/*v*) at 4 °C were added. The solution was stirred and extracted with a homogenizer for 2 min and then centrifuged (10,000 rpm, 4 °C, 50 min) to obtain the supernatant. This was taken as the banana crude extract PE enzyme solution. A total of 15 mL of 0.5% citrus pectin solution containing 0.1 M NaCl was used and adjusted to pH 6.5; next, 1 mL of PE enzyme solution was added and titrated with acid-base [43],

PE activity (Unit) = (A × F × 0.01)/T × 1000 × W(3)

where A = the number of milliliters of 0.01 N sodium hydroxide solution consumed, F = the titer of 0.01 N sodium hydroxide solution, T = the reaction time (minutes), and W = the dilution ratio.

#### 3.2.8. Color Characteristics Analysis

The banana sample was measured with a colorimeter (SA2000, Nippon Denshoku Kogyo Co., Ltd., Tokyo, Japan). The illuminants of the colorimeter were D65, and the observer was a two-degree standard observer for the color analysis of banana peel. The equipment was calibrated before analyzing the sample. The result was L* (lightness), a* (red-green), and b* (yellow-blue) color value representation.

#### 3.2.9. Appearance

At each measurement time point, the bananas of each group were photographed and their appearance changes were recorded.

#### 3.2.10. Scanning Electron Microscopy (SEM) Analysis of D-Limonene Nanoemulsion Coating on Banana Surface

For different concentrations of D-limonene nanoemulsion on bananas, the Guiyi Center of National Sun Yat-sen University (Laboratory A3019, Huan Gong Building and College of Engineering) was entrusted to conduct environmental scanning electron microscope photography to observe the morphology of the bananas’ surface. The accelerating voltage was 10 kV and the amplification was 1000×.

### 3.3. Statistical Analysis

Each measurement was repeated at least three times using different samples. Data were collected and analyzed using one-way ANOVA and Duncan’s test. Significant differences were set at *p* < 0.05. All statistical analyses were performed using the SPSS program (version 12.0, St. Armonk, NY, USA).

## 4. Conclusions

The D-limonene nanoemulsion has good nanoscale and overall particle size uniformity, showing a uniform spherical shape, and its particle size is about 3~5 nm. At the same concentration of D-limonene in nanoemulsion form, it was confirmed that the antibacterial activity could be improved and that it was more effective against Gram-negative bacteria. Bananas are treated with 1.0% and 1.5% D-limonene nanoemulsion coatings, which can reduce the water loss of bananas during storage, reduce the percentage of weight loss, and can also inhibit the activity of pectinase. Bananas are treated with 1.0% and 1.5% D-limonene nanoemulsion coatings, which can delay the generation of total soluble solids and total soluble sugars in bananas. The microbial growth on the surface of bananas treated with 1.0% and 1.5% D-limonene nanoemulsion coatings was significantly inhibited. After the banana was treated with 1.5% D-limonene nanoemulsion coating, the appearance had dark brown lines and shrinkage. Although this has a delaying effect on the after-ripening effect of bananas, it also has a negative appearance. However, this phenomenon did not occur when using a 1.0% D-limonene nanoemulsion coating, which has a good effect and potential on the quality change of bananas during post-harvest storage and shelf–sale extension. This research proved that the D-limonene nanoemulsion coating has an inhibitory effect on the microorganisms on the fruit surface, while maintaining the quality of banana fruit and prolonging the storage life. This material can be applied to other perishable fruit and vegetable products to reduce fruit decay and waste and to improve food safety.

## Figures and Tables

**Table 1:** Determination of the antibacterial activity of D-limonene solution against food-borne pathogens using broth dilution method.

Bacterial	The Real Concentration of D-Limonene Adding in the Medium (%)						
	Control	0.04	0.08	0.1	0.2	0.4	0.8
Log (CFU/mL)							
*Staphylococcus* *aureus*	10.03 ± 0.01 ^a^	9.82 ± 0.04 ^b^	9.33 ± 0.03 ^c^	3.54 ± 0.01 ^d^	ND	ND	ND
*Listeria* *monocytogenes*	9.73 ± 0.04 ^a^	9.58 ± 0.01 ^b^	8.24 ± 0.02 ^c^	7.82 ± 0.00 ^d^	4.48 ± 0.06 ^e^	ND	ND
*Salmonella* *enterica*	10.14 ± 0.02 ^a^	10.00 ± 0.02 ^b^	9.88 ± 0.02 ^c^	9.24 ± 0.01 ^d^	8.21 ± 0.03 ^e^	7.33 ± 0.03 ^f^	ND
*Escherichia* *coli*	10.18 ± 0.18 ^a^	9.40 ± 0.08 ^b^	8.44 ± 0.08 ^c^	8.14 ± 0.08 ^d^	7.86 ± 0.03 ^e^	7.64 ± 0.05 ^e^	ND

ND: not detected. Values within the row followed by the different letters are significantly different (*p* < 0.05). Data are expressed as mean ± SD of triplicate determinations.

**Table 2:** Determination of the antibacterial activity of the D-limonene nanoemulsion against food-borne pathogens using broth dilution method.

Bacterial	The Real Concentration of D-Limonene Adding in the Medium (%)						
	Control	0.04	0.08	0.1	0.2	0.4	0.8
Log (CFU/mL)							
*Staphylococcus* *aureus*	9.22 ± 0.04 ^a^	9.08 ± 0.07 ^b^	4.96 ± 0.11 ^c^	3.59 ± 0.12 ^d^	ND	ND	ND
*Listeria* *monocytogenes*	9.37 ± 0.10 ^a^	9.32 ± 0.03 ^b^	3.12 ± 0.08 ^c^	2.39 ± 0.02 ^d^	ND	ND	ND
*Salmonella* *enterica*	9.45± 0.05 ^a^	9.23 ± 0.16 ^b^	ND	ND	ND	ND	ND
*Escherichia* *coli*	10.49 ± 0.06 ^a^	10.24 ± 0.09 ^b^	7.46 ± 0.16 ^c^	ND	ND	ND	ND

ND: not detected. Values within the row followed by the different letters are significantly different (*p* < 0.05). Data are expressed as mean ± SD of triplicate determinations.

**Table 3:** Values of MIC and MBC concentrations of D-limonene solution and D-limonene nanoemulsion against food-borne pathogens using broth dilution method.

	MIC/MBC (mg/mL)			
Bacterial	D-Limonene Solution		D-Limonene Nanoemulsion	
	MIC	MBC	MIC	MBC
*Staphylococcus* *aureus*	0.336	1.682	0.336	1.682
*Listeria* *monocytogenes*	0.336	3.364	0.336	1.682
*Salmonella* *enterica*	0.336	6.728	0.336	0.673
*Escherichia* *coli*	0.336	6.728	0.336	0.841

**Table 4:** Effect on color characteristics of bananas coated with different concentrations of D-limonene nanoemulsion edible film and stored at 13 ± 1 °C from 0 to 12 days.

Treatment	Storage Time (Days)				
	Day 0	Day 3	Day 6	Day 9	Day 12
L*	58.25 ± 0.83				
Control		67.12 ± 0.61 ^a^	67.33 ± 0.07 ^a^	59.81 ± 1.62 ^b^	49.55 ± 1.17 ^c^
0%		56.43 ± 1.64 ^d^	59.84 ± 2.99 ^b^	59.91 ± 0.20 ^b^	52.44 ± 1.16 ^b^
0.5%		63.84 ± 1.95 ^b^	66.02 ± 0.48 ^a^	59.32 ± 0.63 ^b^	51.82 ± 0.71 ^b^
1.0%		60.93 ± 0.66 ^c^	62.32 ± 0.96 ^b^	62.03 ± 0.82 ^a^	54.32 ± 0.78 ^a^
1.5%		53.95 ± 0.52 ^e^	52.74 ± 0.32 ^c^	51.13 ± 1.30 ^c^	42.80 ± 0.28 ^d^
a*	5.30 ± 0.00				
Control		5.22 ± 0.81 ^a^	6.50 ± 0.88 ^a^	7.89 ± 0.11 ^a^	6.50 ± 0.88 ^a^
0%		−3.27 ± 1.65 ^d^	−1.00 ± 0.47 ^c^	1.44 ± 0.67 ^b^	−1.00 ± 0.47 ^c^
0.5%		3.47 ± 0.64 ^a^	7.35 ± 0.54 ^a^	8.38 ± 0.80 ^a^	7.34 ± 0.54 ^a^
1.0%		−6.86 ± 0.35 ^c^	1.46 ± 1.10 ^b^	7.88 ± 0.33 ^a^	1.46 ± 1.10 ^b^
1.5%		−7.56 ± 1.53 ^c^	−3.38 ± 1.23 ^d^	−2.71 ± 0.50 ^c^	−3.38 ± 1.23 ^d^
b*	45.74 ± 1.35				
Control		46.73 ± 3.72 ^ab^	48.29 ± 1.41 ^c^	45.15 ± 2.35 ^bc^	38.30 ±0.12 ^b^
0%		47.24 ± 0.18 ^ab^	53.04 ± 0.69 ^a^	55.45 ± 0.39 ^a^	46.59 ± 0.97 ^a^
0.5%		49.43 ± 1.07 ^a^	49.98 ± 0.43 ^b^	46.18 ± 1.81 ^b^	37.68 ± 1.54 ^b^
1.0%		42.66 ± 1.30 ^c^	47.00 ± 1.00 ^c^	43.07 ± 1.66 ^bc^	42.99 ± 2.45 ^a^
1.5%		44.47 ± 2.24 ^c^	38.42 ± 0.66 ^c^	41.37 ± 3.42 ^c^	26.18 ± 4.55 ^c^

Data are expressed as mean ± standard deviation from triplicate determination (*n* = 3). Duncan’s test was performed, and the different letters within a column with the same storage time indicate significant differences at the (*p* < 0.05) level.

**Table 5:** Effect on appearance changes of banana coated with different concentrations of D-limonene nanoemulsion edible film and stored at 13 ± 1 °C from 0 to 12 days.

	Control	0%	0.5%	1.0%	1.5%
Day 0	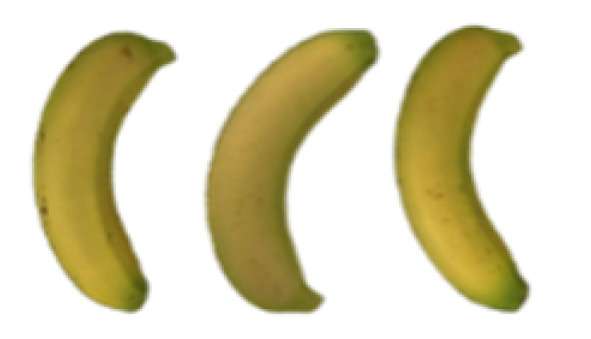	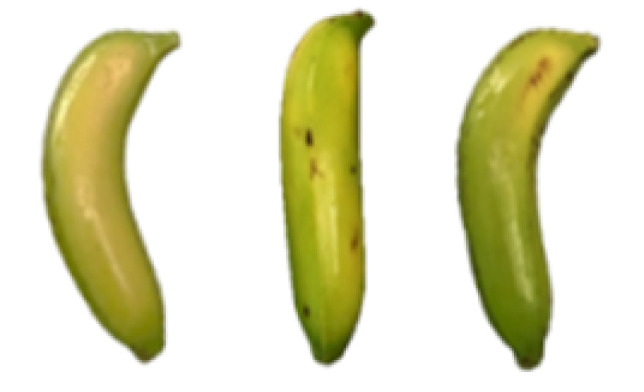	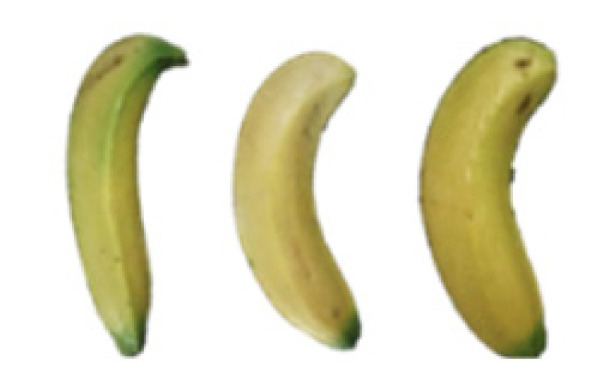	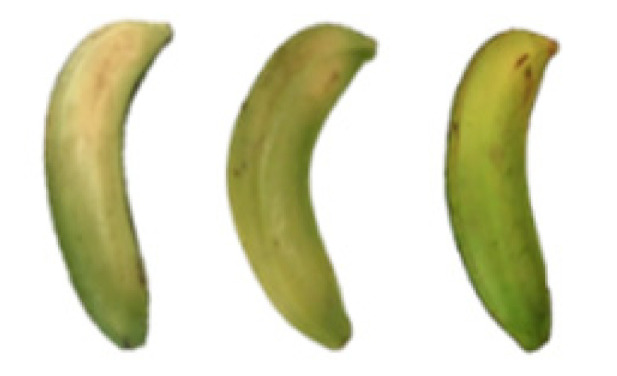	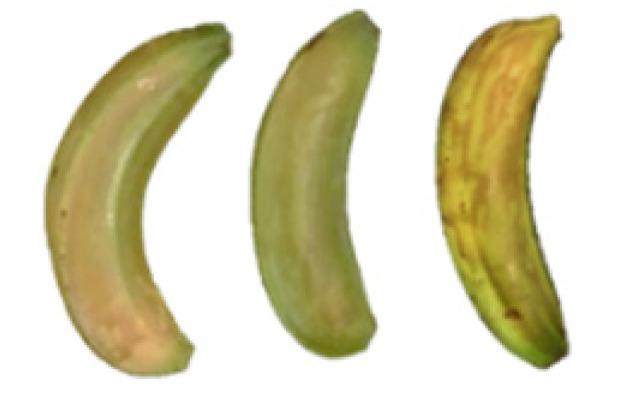
Day 3	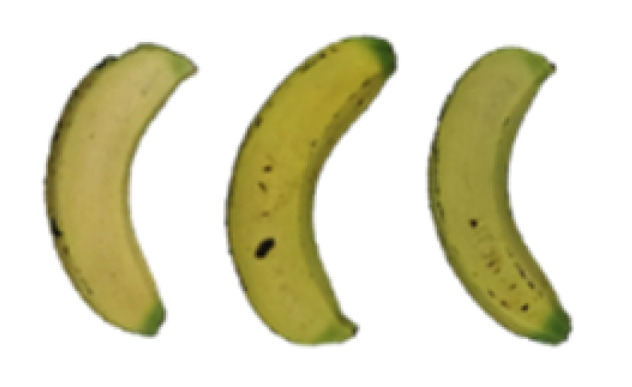	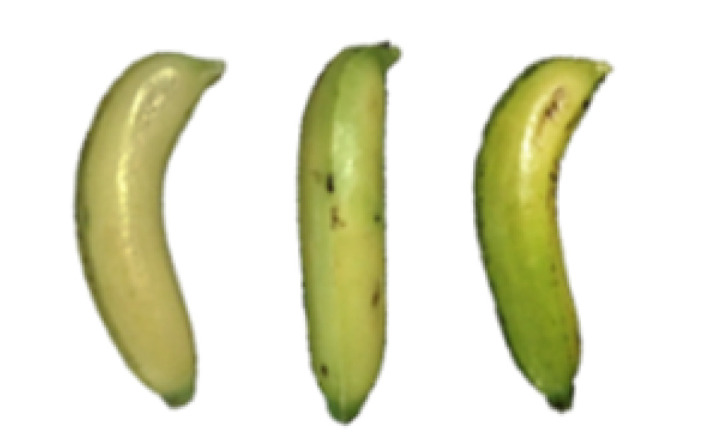	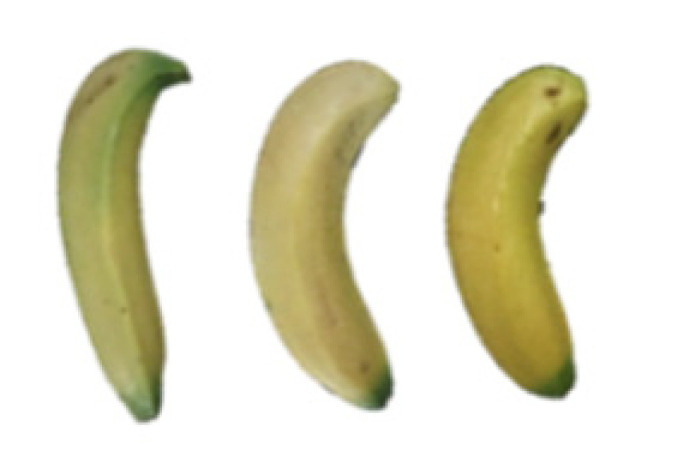	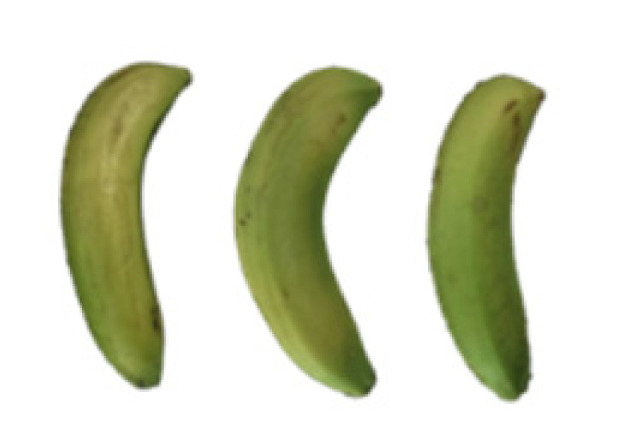	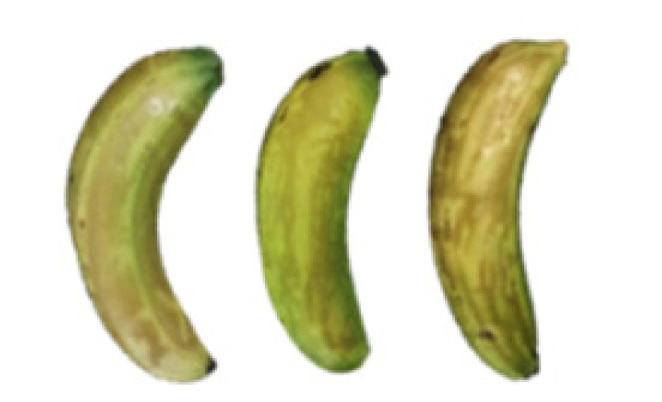
Day 6	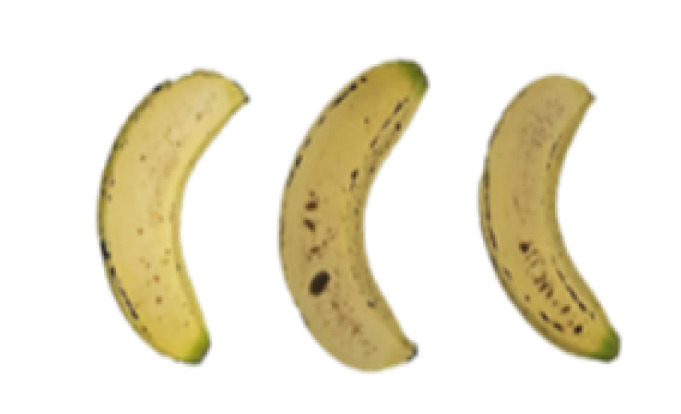	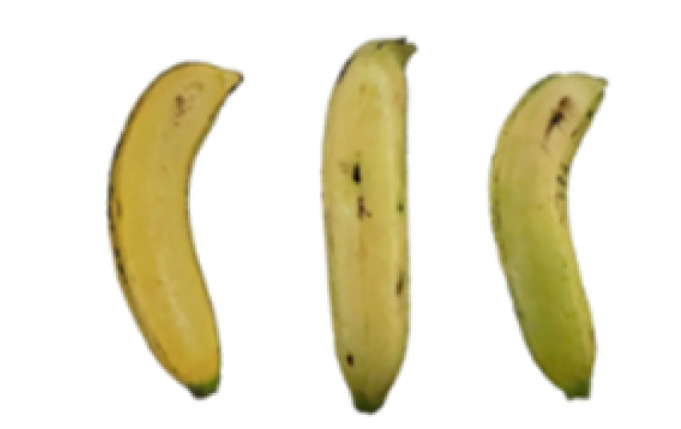	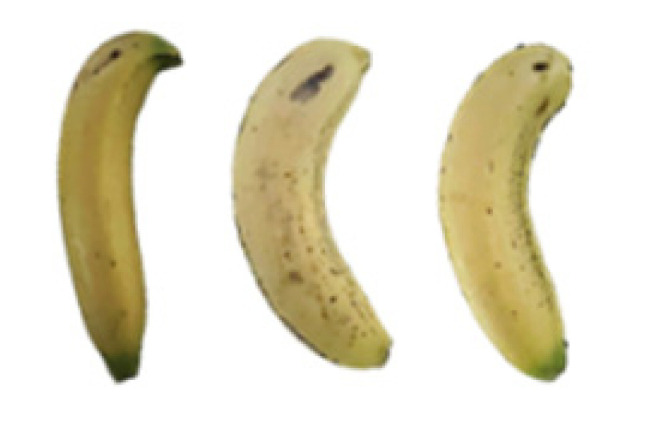	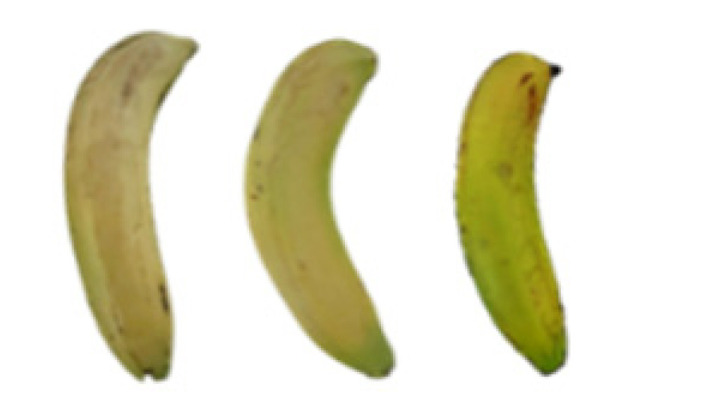	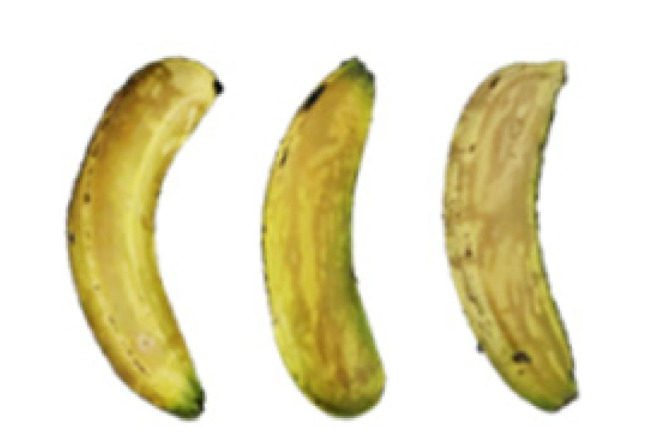
Day 9	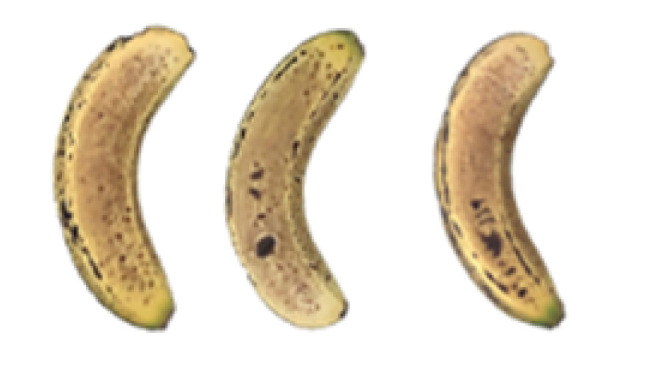	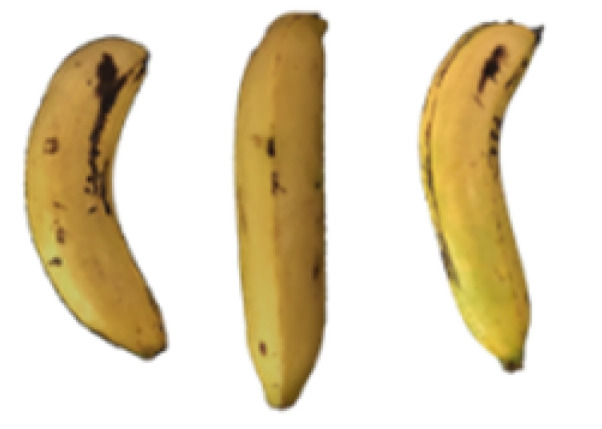	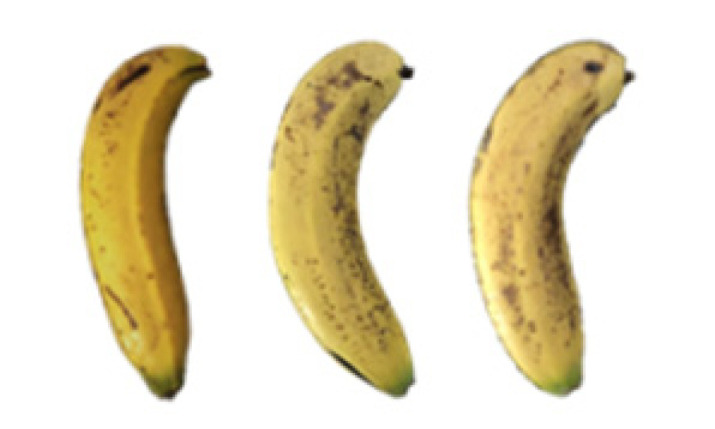	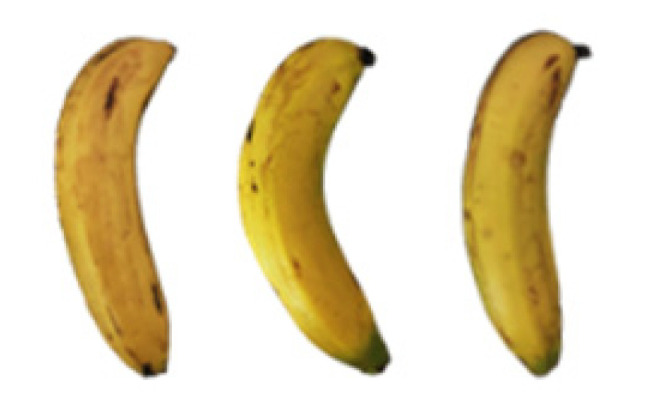	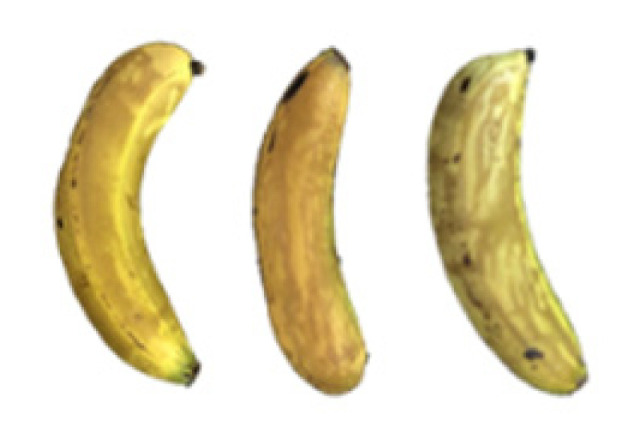
Day 12	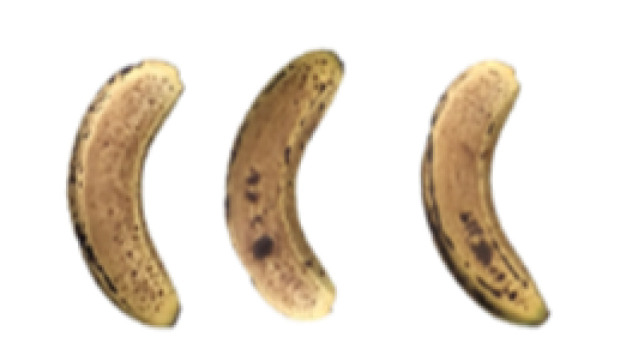	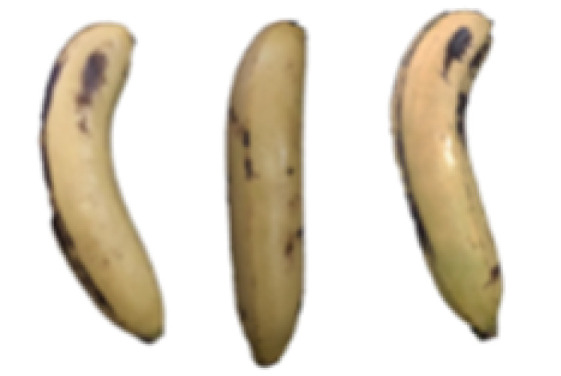	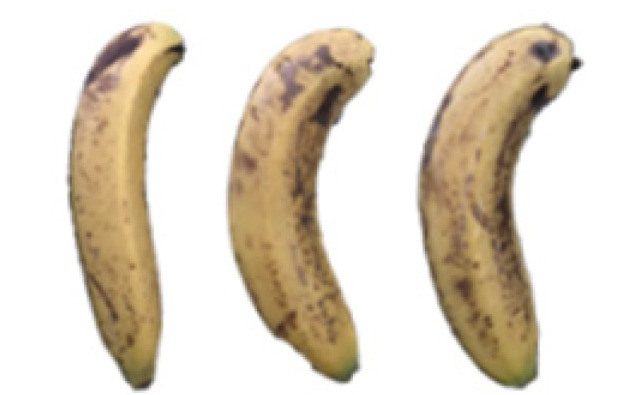	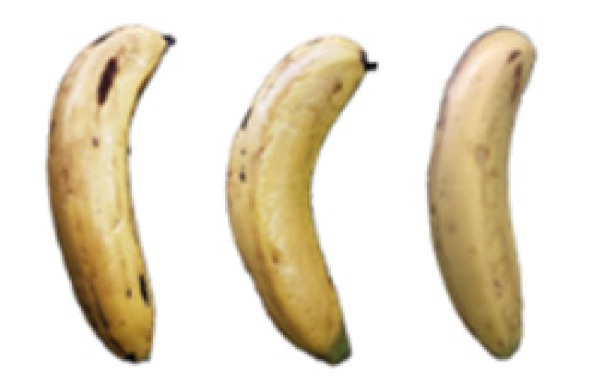	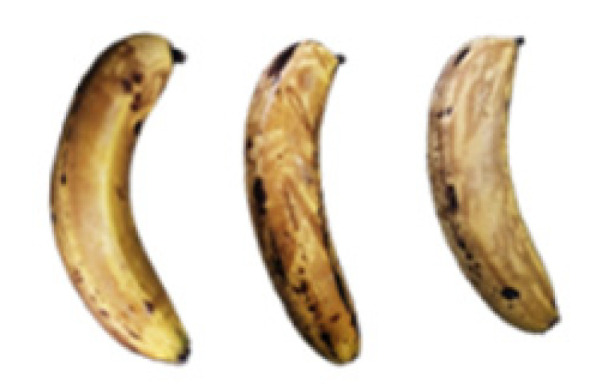

## Data Availability

Not applicable.
